# Outcomes and risk factors of hemorrhage in patients with resected brain metastases

**DOI:** 10.1002/ijc.70250

**Published:** 2025-12-07

**Authors:** Melisa S. Guelen, Kiarash Ferdowssian, Niklas Jung, Hava N. Celik, Andrea Dell'Orco, Semil Eminovic, Anton Früh, Majd Samman, Güliz Acker, Arend Koch, Helena Radbruch, Michael Scheel, Mike P. Wattjes, Julia Onken, Peter Vajkoczy, Nils Hecht, Jawed Nawabi, David Wasilewski

**Affiliations:** ^1^ Department of Neurosurgery Charité – Universitätsmedizin Berlin, Corporate Member of Freie Universität Berlin and Humboldt‐Universität zu Berlin Berlin Germany; ^2^ Institute of Neuroradiology Charité – Universitätsmedizin Berlin, Corporate Member of Freie Universität Berlin and Humboldt‐Universität zu Berlin Berlin Germany; ^3^ Athinoula A. Martinos Center for Biomedical Imaging Massachusetts General Hospital and Harvard Medical School Charlestown Massachusetts USA; ^4^ Department of Radiology Charité – Universitätsmedizin Berlin, Corporate Member of Freie Universität Berlin and Humboldt‐Universität zu Berlin Berlin Germany; ^5^ BIH Biomedical Innovation Academy, BIH Charité Junior Digital Clinician Scientist Program Berlin Institute of Health at Charité – Universitätsmedizin Berlin Berlin Germany; ^6^ Department of Neuropathology Charité – Universitätsmedizin Berlin, Corporate Member of Freie Universität Berlin and Humboldt‐Universität zu Berlin Berlin Germany; ^7^ German Cancer Consortium (DKTK) Partner Site Berlin and German Cancer Research Center (DKFZ) Heidelberg Germany; ^8^ Department of Neurosurgery, Faculty of Medicine and University Hospital Düsseldorf Heinrich‐Heine University Düsseldorf Düsseldorf Germany

**Keywords:** anticoagulants, hemorrhagic brain metastasis, intracranial hemorrhage, melanoma, survival

## Abstract

Brain metastases (BrMs) may present with intralesional or intracranial hemorrhage (ICH), yet risk factors and outcomes remain unclear. This monocentric cohort study at Germany's largest neurosurgical clinic included 973 adults undergoing BrM resection (2010–2024), with histopathologically confirmed etiologies and known tumor burden. Based on pre‐operative CT or MRI, 880 patients were categorized as non‐hemorrhagic (non‐hBrM), presenting with intralesional hemorrhage (hBrM), or with ICH of ≥30 mm diameter (ICH‐BrM). Risk factors for hBrM and ICH‐BrM were assessed, and overall survival (OS) and progression‐free survival (PFS) were analyzed using Kaplan‐Meyer methods. Of 880 patients, 560 (63.6%) were non‐hBrM, 243 (27.6%) hBrM, and 77 (8.8%) ICH‐BrM. ICH‐BrM had larger tumor volume (21 cm^3^, IQR 13–34) than hBrM (14 cm^3^, IQR 6–28) and non‐hBrM (12 cm^3^, IQR 6–21) (*p*_adjust_ = .017), correlated with lower post‐op Karnofsky index (*p*_adjust_ = .047), dsGPA score (*p*_adjust_ = .032), and more BrMs (*p*_adjust_ = .004). Pre‐operative antithrombotic use did not differ between groups (*p*_adjust_ = .32). Melanoma was more common in hBrM (27.8%) and ICH‐BrM (38.0%), predicting ICH (OR 2.95, *p* < .001) along with NSCLC (OR 1.64, *p* < .001). ICH did not independently predict worse OS (HR 1.23, *p* = .38). Worse OS was linked to larger tumor volume (HR 1.35, *p* = .002), extracranial metastases (HR 1.77, *p* < .001), and older age (HR 1.53, *p* < .001), while KPS >80% (HR 0.77, *p* < .01), solitary BrM (HR 0.62, *p* = .002), and adjuvant treatments (*p* < .001) predicted improved OS. ICH is associated with larger tumors and melanoma but is not an independent OS predictor. Tumor burden, extracranial metastases, and adjuvant treatments drive BrM survival.

## What's New?

Brain metastases may present with hemorrhage, yet risk factors and clinical outcomes remain unclear. Based on a cohort of 880 patients with resected brain metastases, this study identified tumor volume and primary tumor histology (melanoma and non‐small cell lung cancer) as key predictors of hemorrhage. Notably, antithrombotic medication was not a significant risk factor. Patient functional status and adjuvant treatments predicted improved overall survival. Hemorrhage did not independently predict overall survival, emphasizing the dominance of tumor burden, extracranial metastases, and adjuvant treatments in driving outcomes. These findings provide critical insights for guiding the clinical management of hemorrhagic brain metastases.

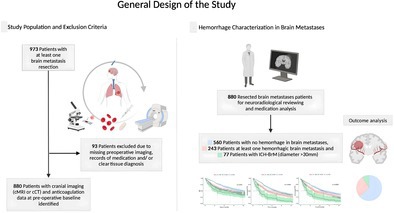

**Abbreviations**
ADC: apparent diffusion coefficient
BrMs: brain metastases
cCT: cranial computed tomography
CI: confidence intervals
cMRI: cranial magnetic resonance imaging
dsGPA: diagnosis‐specific graded prognostic assessment
DTI: diffusion tensor imaging
DWI: diffusion‐weighted imaging
ecPFS: extracranial progression‐free survival
hBrM: brain metastases with intralesional hemorrhage (cf. text)
HR: hazard ratios
ICH: intracranial hemorrhage
ICH‐BrM: brain metastases with ICH of ≥30 mm diameter (cf. text)
icPFS: intracranial progression‐free survival
IQR: interquartile range
iRANO: immunotherapy response assessment for neuro‐oncology
KPS: Karnofsky performance score
MPRAGE: magnetization prepared rapid gradient echo
non‐hBrM: non‐hemorrhagic brain metastases (cf. text)
NSCLC: non‐small cell lung cancer
OS: overall survival
PACS: picture archiving and communication system
PFS: progression‐free survival
RECIST: response evaluation criteria in solid tumors
SWI: susceptibility weighted imaging
T2w: T2‐weighted (imaging)
T2‐w FLAIR: T2‐weighted‐fluid‐attenuated inversion recovery

## 1 INTRODUCTION

Brain metastases (BrMs) are the most common intracranial tumors in adults, primarily arising from lung, breast, melanoma, and renal cell cancers. Advances in imaging and oncological therapies have increased the detection and management of BrMs, affecting up to 40% of patients with solid malignancies during their disease progression.^1^, ^2^ Certain tumor types, including melanoma and renal cell carcinoma, are more prone to hemorrhagic complications, either presenting with signs of intralesional hemorrhage (hBrM) or bona fide intracranial hemorrhage (ICH‐BrM), which can result in significant neurological deficits.^3^, ^4^, ^5^, ^6^, ^7^ Neurosurgical resection remains a viable treatment.^7^

Despite the clinical impact of hemorrhagic brain metastases, their incidence, risk factors, and prognostic implications remain poorly defined.^8^, ^9^, ^10^, ^11^ Studies on therapeutic antithrombotic medication have demonstrated that these treatments, including anticoagulation and antiplatelet treatment, are not associated with increased risk of ICH‐BrM.^6^, ^7^, ^12^, ^13^, ^14^, ^15^ Larger tumor size, melanoma histology, and combined therapies involving tyrosine kinase inhibitors and radiation therapy, on the other hand, have been associated with an elevated risk of hemorrhage.^6^, ^7^ However, existing studies often rely on small cohorts or lack comprehensive imaging and histopathological data, limiting their generalizability.

This prospective and retrospective cohort analysis study aimed to evaluate risk factors and outcomes associated with hBrM and ICH‐BrM in a large cohort of 880 patients with histopathologically confirmed BrMs from our brain metastasis registry. By integrating data from various cancer types and analyzing clinical, imaging, and pre‐operative factors such as tumor volume and use of antithrombotic medication, we sought to clarify the prognostic significance of hemorrhagic complications for overall survival (OS) and progression‐free survival (PFS), and provide evidence to guide clinical management.

## 2 METHODS

### 2.1 Patient cohort

This retrospective and partially prospective study included 880 patients with resected brain metastases from January 2010 to July 2024. Of these, 724 patients were included retrospectively, and 156 were prospectively recruited.

### 2.2 Cohort definition, inclusion and exclusion criteria

**FIGURE 1:**
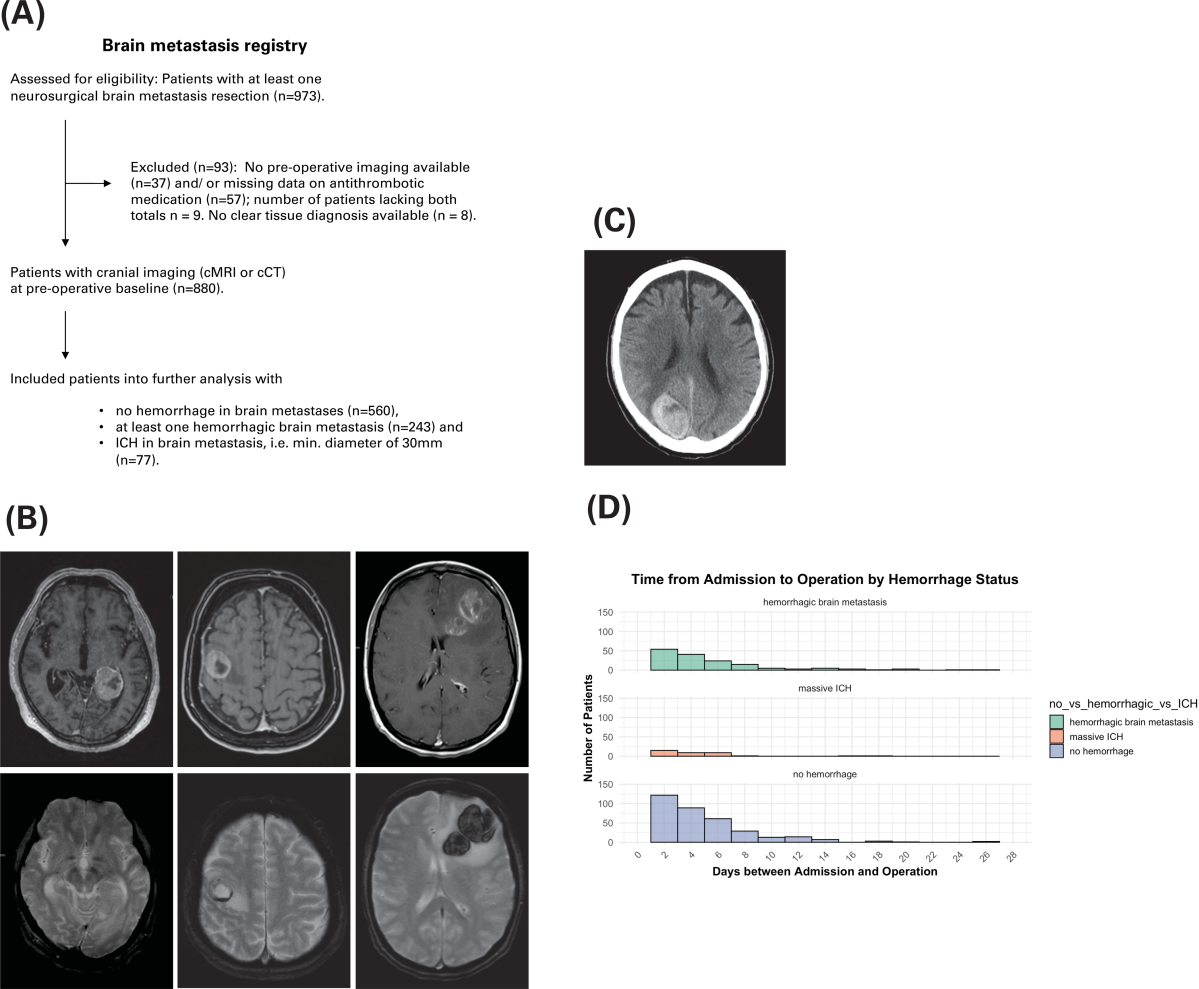
CONSORT diagram representing the study population with excluded and included patients. Our cohort involved in total 973 patients that underwent at least one neurosurgical brain metastasis resection, from which 88 patients were excluded due to lack of pre‐operative imaging and/or missing data on antithrombotic medication or lack of clear tissue diagnosis. The final cohort includes patients with no hemorrhage in brain metastases (*n* = 560), at least one hemorrhagic brain metastasis (*n* = 243) or BrM‐associated ICH, i.e. min. diameter 30 mm (*n* = 77). (B) Axial cMRI slices with brain metastases from three patients from our cohort, representative of non‐hemorrhagic BrM, hemorrhagic BrM, and ICH‐BrM. Upper row: Post‐contrast T1‐weighted images. Lower row: according T2*‐weighted images aligned in the same slice. This composite illustrates the radiological profiles of brain metastases in our cohort, showcasing distinct characteristics between T1 and T2*‐weighted imaging modalities: Each column represents a patient, exemplifying the spectrum of bleeding behavior of central metastatic lesions by representing the three categories observed within our cohort: f.l.t.r. patient 1 (NSCLC BrM): non‐hemorrhagic BrM; patient 2 (malignant melanoma BrM): hemorrhagic BrM; patient 3 (NSCLC BrM): BrM‐ICH. (C) Axial cCT scan screenshot of BrM‐associated ICH in right occipital lobe. (D) Time from admission to operation by hemorrhage status. This bar chart shows the time in days between admission and operation for the three patient groups.

The baseline was defined as the date of primary resection of a brain metastasis for each patient. As described in the CONSORT diagram (Figure 1A), imaging data were screened for a total of 973 patients. Images were identified on the local picture archiving and communication system (PACS) server and pulled into a local container for systemic imaging analysis. Two raters (D.W., 5 years and M.G., 2 years of experience) reviewed the latest cranial imaging study prior to the baseline for each patient, that is, cranial magnetic resonance imaging (cMRI) in axial plane, with all available sequences of the standard local cMRI protocol (consisting of the following: coronal T2‐weighted‐fluid‐attenuated inversion recovery (T2‐w FLAIR), diffusion‐weighted imaging (DWI), apparent diffusion coefficient (ADC) with additional diffusion tensor imaging (DTI), T1‐weighted imaging before and after contrast enhancement, 3D T1‐weighted post‐contrast MP‐RAGE, and either T2*‐weighted imaging (T2w*) or susceptibility weighted imaging (SWI) sequences and 3D T1w post‐contrast MPRAGE (magnetization prepared rapid gradient echo) including hemosiderin‐sensitive sequences (T2w* or SWI) and/or cranial computed tomography (cCT) in axial plane to detect signs of intralesional hemorrhage or ICH^14^) (Figure 1B,C). MRI data were heterogeneous with respect to field strength (1.5 and 3 T), MRI manufacturer (Siemens, Erlangen, Germany; General Electric Healthcare, Milwaukee, USA), MRI protocol, and contrast agent (gadoterate meglumine, gadobutrol). Yet, all MRI studies included at least axial T1‐weighted, T2‐weighted, and T2‐w FLAIR‐series. If both cMRI and cCT study types were available before baseline, both were analyzed. In the case of ICH‐BrM, not all patients had a cMRI before surgery, given that these patients frequently underwent surgical emergency evacuation of their index lesion, which is why it was not always possible to compare hemorrhage sizes using the exact same imaging modalities. The volumetric accuracy of different imaging modalities in acute intracerebral hemorrhage and comparisons between CT and MRI modalities for emergency stroke assessments have been investigated in previous studies.^16^, ^17^ In the case of a known metastasis due to a melanoma, the hemorrhagic event was further assessed using susceptibility infarcts on contrast enhanced imaging.

Further analysis of the baseline included reviewing patient encounter reports and electronic files on the local patient information systems to assess pre‐operative antithrombotic medication intake. Finally, after excluding patients with a lack of pre‐operative cMRI and/or cCT scans, antithrombotic medication data prior to brain metastasis resection and/or clear histopathological brain metastasis tissue diagnosis, 880 patients were included (Figure 1A).

Patients were categorized based on pre‐operative cranial imaging findings into three groups:

1. Patients without signs of hemorrhage on cranial imaging (non‐hemorrhagic BrM, henceforth referred to as non‐hBrM), that is, in these patients, no signs of hemorrhage were visible neither on cMRI with T2* or SWI nor on cCT;
2. Patients with at least one brain metastasis with signs of intralesional hemorrhage (hBrM) on cranial imaging, that is, on cMRI with T2* or SWI or on cCT (arbitrary maximal diameter of index lesion set <30 mm);
3. Patients with classic (space‐occupying) intracranial hemorrhage (ICH‐BrM) (arbitrary lesion diameter cut‐off set to 30 mm) measured on the axial slice of cMRI (T2w* or SWI) or cCT study on which ICH presents with largest diameter prior to baseline, measured using the PACS ruler tool.

The raters were blinded with the reports to exclude any bias and only after assessment of the images were the results confirmed with the radiology report of the respective scans. In case of discrepancies or if the image analysis and reports did not allow for a certain categorization, especially when patients were initially classified as hemorrhagic, a consensus was conducted by board‐certified neuroradiologist J.N. (9 years of expertise in ICH imaging) for a total of 211 patients.

### 2.3 Additional Radiological Data

Tumor volumes of index brain metastatic lesions and associated edema volumes of the index metastasis, that is, resected brain metastasis, were quantified using a semiautomated 3‐dimensional rendering algorithm in iPlannet (Brainlab) using the SmartBrush tool (T1‐weighted images for tumor measurements and fluid‐attenuated inversion recovery images for edema measurements). Tumor volumes were then dichotomized, where cut‐offs were determined via log‐rank analysis^18^ (tumor volume: 11.4 cm^3^; edema volume: 27.8 cm^3^) (Figure S1A,B). The localization (supratentorial, infratentorial or both) and anatomical region (frontal, temporal, parietal, occipital, cerebellar or other) of the index brain metastasis as well as the number of brain metastases as a categorical value (brain metastasis burden as 1 brain metastasis, 2 brain metastases or ≥2 brain metastases at baseline or absolute number of brain metastases at baseline) were derived from the imaging studies. The presence of extracranial metastases was assessed by means of CT staging within 8 weeks of primary brain metastasis resection.

### 2.4 Clinical and outcome data

Clinical data retrieved from clinical patient records encompassed demographics, the leading symptom before primary brain metastasis resection, Karnofsky Performance Score (KPS), post‐operative diagnosis‐specific graded prognostic assessment (ds‐GPA), pre‐operative treatments including concurrent antithrombotic medication (vitamin K or non‐vitamin K oral anticoagulants, low‐molecular‐weight heparins or antiplatelet agents such as aspirin or clopidogrel), time of admission, time of primary brain metastasis resection and post‐operative treatment regimens (best supportive care vs. local therapy only, i.e., post‐operative radiation therapy vs. postoperative radiation therapy with systemic therapy, i.e., chemotherapy, radiation therapy, immune checkpoint inhibition therapy or targeted therapy) (Figure 1D and Tables 1A, 1B, 1C). The distribution of leading symptoms before primary brain metastasis resection was further analyzed by comparing frequencies of the individual symptoms, and by comparing grouped symptom complexes as binary symptoms, namely focal neurological deficits (FND), including hemiparesis (motor deficit), sensory deficit, aphasia, visual impairment, vertigo or cerebellar signs, and increased intracranial pressure (ICP) including headache, nausea, or vomiting.

**TABLE 1A:** Comparison of patient characteristics between non‐hemorrhagic brain metastases, hemorrhagic brain metastases, and patients with brain metastasis‐associated ICH.

Variable	*N*	hemorrhagic brain metastasis, *N* = 243	ICH, *N* = 77	No hemorrhage, *N* = 560	*p*‐value^a^	*q*‐value^b^
Age, median (IQR)	849	66 (55–73)	63 (56–73)	63 (55–72)	.29	0.32
Sex, *n* (%)	880				.005	0.012
Female		103 (42)	40 (52)	307 (55)		
Male		140 (58)	37 (48)	253 (45)		
KPS group, *n* (%)	799				.030	0.047
<80%		91 (44)	23 (58)	209 (38)		
80–100%		118 (56)	17 (43)	341 (62)		
GPA group, *n* (%)	777				.019	0.032
>2		78 (38)	9 (26)	247 (46)		
0–2		128 (62)	25 (74)	290 (54)		
Extracranial metastases at baseline, *n* (%)	794	117 (56)	28 (72)	240 (44)	<.001	<0.001
Underlying tumor entity, *n* (%)	880				<.001	<0.001
Breast cancer		17 (7.0)	5 (6.5)	115 (21)		
Melanoma		68 (28)	30 (39)	43 (7.7)		
NSCLC		106 (44)	17 (22)	299 (53)		
Other		52 (21)	25 (32)	103 (18)		
Tumor volume, median (IQR)	697	14 (6–28)	21 (13–34)	12 (6–21)	.009	0.017
Edema volume, median (IQR)	661	64 (37–112)	73 (34–109)	59 (29–111)	.48	0.48
Anatomical site, *n* (%)	880					
Cerebellar		41 (17)	18 (23)	152 (27)		
Frontal		80 (33)	24 (31)	178 (32)		
Occipital		34 (14)	8 (10)	57 (10)		
Other		8 (3.3)	0 (0)	13 (2.3)		
Parietal		37 (15)	19 (25)	97 (17)		
Temporal		43 (18)	8 (10)	63 (11)		
Leading symptom, *n* (%)	880					
Alterations in behavior, disorientation, aphasia		21 (8.6)	13 (17)	62 (11)		
Cerebellar symptoms		10 (4.1)	10 (13)	38 (6.8)		
Classic HNV (headache, nausea, vomiting)		33 (14)	3 (3.9)	100 (18)		
During staging		17 (7.0)	5 (6.5)	34 (6.1)		
Headache		27 (11)	5 (6.5)	56 (10)		
Incidental		7 (2.9)	1 (1.3)	36 (6.4)		
Others		1 (0.4)	0 (0)	0 (0)		
Seizures		18 (7.4)	5 (6.5)	59 (11)		
Sensory–motor symptoms or hemiparesis		55 (23)	22 (29)	94 (17)		
Unknown		14 (5.8)	2 (2.6)	9 (1.6)		
Vertigo and dyscoordination		24 (9.9)	5 (6.5)	38 (6.8)		
Visual impairment		16 (6.6)	6 (7.8)	34 (6.1)		
Hydrocephalus at baseline, *n* (%)	822				.26	0.31
Hydrocephalus		19 (8.6)	5 (11)	71 (13)		
No hydrocephalus		201 (91)	41 (89)	485 (87)		
Total number categorized, *n* (%)	864				.009	0.017
≥2 brain metastases at baseline		94 (39)	30 (40)	151 (27)		
1 brain metastasis at baseline		79 (33)	26 (35)	227 (41)		
2 brain metastases at baseline		66 (28)	19 (25)	172 (31)		
Absolute number, median (IQR)	626	1.00 (1.00–3.00)	2.00 (1.00–3.00)	1.00 (1.00–2.00)	.001	0.004
Localization, *n* (%)	848				.34	0.36
Both		45 (19)	18 (24)	121 (22)		
Infratentorial		31 (13)	11 (15)	97 (18)		
Supratentorial		156 (67)	45 (61)	324 (60)		
Pre‐treatment status before primary brain metastasis resection, *n* (%)	828				.079	0.11
Pre‐treated before brain metastasis resection		101 (44)	40 (60)	251 (47)		
Treatment‐naive		128 (56)	27 (40)	281 (53)		
Adjuvant treatment after primary brain metastasis resection, *n* (%)	714				<.001	<0.001
Best supportive care		57 (28)	24 (38)	52 (12)		
Radiation therapy		38 (19)	12 (19)	120 (27)		
Radiation therapy and chemotherapy		43 (21)	6 (9.4)	133 (30)		
Radiation therapy and immunotherapy		50 (25)	20 (31)	93 (21)		
Radiation therapy and targeted therapy		13 (6.5)	2 (3.1)	51 (11)		
da, *n* (%)	880	51 (21)	23 (30)	136 (24)	.26	0.31
Ki67 status, *n* (%)	756				.048	0.070
<30%		120 (57)	44 (67)	246 (51)		
≥30%		92 (43)	22 (33)	232 (49)		
Survival, median (IQR)	878	6 (2–21)	3 (1–10)	11 (4–25)	<.001	<0.001
icPFS, median (IQR)	798	4 (2–12)	2 (1–6)	6 (3–14)	<.001	<0.001
ecPFS, median (IQR)	880	3 (0–10)	0 (0–2)	5 (1–14)	<.001	<0.001

*Note*: This table presents the comparison of key clinical characteristics between three groups of patients: those with non‐hemorrhagic brain metastases, those with hemorrhagic brain metastases, and patients with brain metastasis‐associated ICH. The variables include demographic data (e.g., age, sex), clinical performance scores (KPS, GPA), tumor‐associated characteristics (volume, edema volume), and treatment details (e.g., adjuvant therapies, pre‐operative anticoagulation). *p*‐values are provided to indicate the statistical significance of differences between the groups, with correction for multiple testing using *q*‐values (or adjusted *p*‐values).

^a^ Kruskal–Wallis rank sum test; Pearson's Chi‐squared test.

^b^ False discovery rate correction for multiple testing.

**TABLE 1B:** Comparison of patient characteristics between non‐hemorrhagic brain metastases vs. patients with hemorrhagic brain metastasis or brain metastasis‐associated ICH.

Variable	*N*	No hemorrhagic brain metastasis, *N* = 560	Hemorrhagic brain metastases or ICH, *N* = 320	*p*‐value^a^	*q*‐value^b^
Age, median (IQR)	849	63 (55–72)	65 (55–73)	.16	0.20
Sex, *n* (%)	880			.004	0.008
Female		307 (55)	143 (45)		
Male		253 (45)	177 (55)		
KPS group, *n* (%)	799			.038	0.061
<80%		209 (38)	114 (46)		
80–100%		341 (62)	135 (54)		
GPA group, *n* (%)	777			.011	0.021
>2		247 (46)	87 (36)		
0–2		290 (54)	153 (64)		
Extracranial metastasis at baseline, *n* (%)	794	240 (44)	145 (58)	<.001	<0.001
Underlying tumor entity, *n* (%)	880			<.001	<0.001
Breast cancer		115 (21)	22 (6.9)		
Melanoma		43 (7.7)	98 (31)		
NSCLC		299 (53)	123 (38)		
Other		103 (18)	77 (24)		
Tumor volume, median (IQR)	697	12 (6–21)	15 (6–31)	.034	0.059
Edema volume, median (IQR)	661	59 (29–111)	69 (37–109)	.24	0.26
Anatomical site, *n* (%)	880			.042	0.063
Cerebellar		152 (27)	59 (18)		
Frontal		178 (32)	104 (33)		
Occipital		57 (10)	42 (13)		
Other		13 (2.3)	8 (2.5)		
Parietal		97 (17)	56 (18)		
Temporal		63 (11)	51 (16)		
Leading symptom, *n* (%)	880				
Alterations in behavior, disorientation, aphasia		62 (11)	34 (11)		
Cerebellar symptoms		38 (6.8)	20 (6.3)		
Classic HNV (headache, nausea, vomiting)		100 (18)	36 (11)		
During staging		34 (6.1)	22 (6.9)		
Headache		56 (10)	32 (10)		
Incidental		36 (6.4)	8 (2.5)		
Others		0 (0)	1 (0.3)		
Seizures		59 (11)	23 (7.2)		
Sensory–motor symptoms or hemiparesis		94 (17)	77 (24)		
Unknown		9 (1.6)	16 (5.0)		
Vertigo and dyscoordination		38 (6.8)	29 (9.1)		
Visual impairment		34 (6.1)	22 (6.9)		
Hydrocephalus at baseline, *n* (%)	822			.12	0.15
Hydrocephalus		71 (13)	24 (9.0)		
No hydrocephalus		485 (87)	242 (91)		
Bleeding group, *n* (%)	880			<.001	<0.001
Hemorrhagic brain metastasis		0 (0)	243 (76)		
ICH		0 (0)	77 (24)		
No hemorrhage		560 (100)	0 (0)		
Total number categorized, *n* (%)	864			.001	0.003
≥2 brain metastases at baseline		151 (27)	124 (39)		
1 brain metastasis at baseline		227 (41)	105 (33)		
2 brain metastases at baseline		172 (31)	85 (27)		
Absolute number, median (IQR)	626	1.00 (1.00–2.00)	1.00 (1.00–3.00)	<.001	0.001
Localization, *n* (%)	848			.18	0.21
Both		121 (22)	63 (21)		
Infratentorial		97 (18)	42 (14)		
Supratentorial		324 (60)	201 (66)		
Pre‐treatment status before primary brain metastasis resection, *n* (%)	828			.90	0.90
Pre‐treated before brain metastasis resection		251 (47)	141 (48)		
Treatment‐naive		281 (53)	155 (52)		
Adjuvant treatment after primary brain metastasis resection, *n* (%)	714			<.001	<0.001
Best supportive care		52 (12)	81 (31)		
Radiation therapy		120 (27)	50 (19)		
Radiation therapy and chemotherapy		133 (30)	49 (18)		
Radiation therapy and immunotherapy		93 (21)	70 (26)		
Radiation therapy and targeted therapy		51 (11)	15 (5.7)		
Pre‐operative antithrombotic intake, *n* (%)	880	136 (24)	74 (23)	.70	0.73
Ki67 status, *n* (%)	756			.045	0.063
<30%		246 (51)	164 (59)		
≥30%		232 (49)	114 (41)		
Survival, median (IQR)	878	11 (4–25)	5 (2–19)	<.001	<0.001
icPFS, median (IQR)	798	6 (3–14)	4 (2–11)	.004	0.008
ecPFS, median (IQR)	880	5 (1–14)	2 (0–8)	<.001	<0.001

*Note*: This table compares patients with non‐hemorrhagic brain metastases against those with either hemorrhagic brain metastasis or brain metastasis‐associated ICH. The table includes patient characteristics such as age, sex, performance status (KPS, GPA), tumor volume, extracranial metastasis, and adjuvant treatment details. Statistical comparisons using *p*‐values and *q*‐values highlight the significant differences between these groups, indicating factors that may differentiate hemorrhagic events.

^a^ Wilcoxon rank sum test; Pearson's Chi‐squared test.

^b^ False discovery rate correction for multiple testing.

**TABLE 1C:** Comparison of patient characteristics between non‐hemorrhagic brain metastases and patients with hemorrhagic brain metastasis vs. brain metastasis‐associated ICH.

Variable	*N*	ICH, *N* = 77	No ICH, *N* = 803	*p*‐value^a^	*q*‐value^b^
Age, median (IQR)	849	63 (56–73)	64 (55–72)	.89	0.89
Sex, *n* (%)	880			.88	0.89
Female		40 (52)	410 (51)		
Male		37 (48)	393 (49)		
KPS group, *n* (%)	799			.024	0.050
<80%		23 (58)	300 (40)		
80–100%		17 (43)	459 (60)		
GPA group, *n* (%)	777			.047	0.075
>2		9 (26)	325 (44)		
0–2		25 (74)	418 (56)		
Extracranial metastasis at baseline, *n* (%)	794	28 (72)	357 (47)	.003	0.008
Underlying tumor entity, *n* (%)	880			<.001	<0.001
Breast cancer		5 (6.5)	132 (16)		
Melanoma		30 (39)	111 (14)		
NSCLC		17 (22)	405 (50)		
Other		25 (32)	155 (19)		
Tumor volume, median (IQR)	697	21 (13–34)	12 (6–23)	.005	0.013
Edema volume, median (IQR)	661	73 (34–109)	61 (31–111)	.50	0.62
Anatomical site, *n* (%)	880			.48	0.62
Cerebellar		18 (23)	193 (24)		
Frontal		24 (31)	258 (32)		
Occipital		8 (10)	91 (11)		
Other		0 (0)	21 (2.6)		
Parietal		19 (25)	134 (17)		
Temporal		8 (10)	106 (13)		
Leading symptom, *n* (%)	880				
Alterations in behavior, disorientation, aphasia		13 (17)	83 (10)		
Cerebellar symptoms		10 (13)	48 (6.0)		
Classic HNV (headache, nausea, vomiting)		3 (3.9)	133 (17)		
During staging		5 (6.5)	51 (6.4)		
Headache		5 (6.5)	83 (10)		
Incidental		1 (1.3)	43 (5.4)		
Others		0 (0)	1 (0.1)		
Seizures		5 (6.5)	77 (9.6)		
Sensory–motor symptoms or hemiparesis		22 (29)	149 (19)		
Unknown		2 (2.6)	23 (2.9)		
Vertigo and dyscoordination		5 (6.5)	62 (7.7)		
Visual impairment		6 (7.8)	50 (6.2)		
Hydrocephalus at baseline, *n* (%)	822			.88	0.89
Hydrocephalus		5 (11)	90 (12)		
No hydrocephalus		41 (89)	686 (88)		
Total number categorized, *n* (%)	864			.28	0.39
≥2 brain metastases at baseline		30 (40)	245 (31)		
1 brain metastasis at baseline		26 (35)	306 (39)		
2 brain metastases at baseline		19 (25)	238 (30)		
Absolute number, median (IQR)	626	2.00 (1.00–3.00)	1.00 (1.00–2.00)	.020	0.046
Localization, *n* (%)	848			.82	0.89
Both		18 (24)	166 (21)		
Infratentorial		11 (15)	128 (17)		
Supratentorial		45 (61)	480 (62)		
Pre‐treatment status before primary brain metastasis resection, *n* (%)	828			.035	0.061
Pre‐treated before brain metastasis resection		40 (60)	352 (46)		
Treatment‐naive		27 (40)	409 (54)		
Adjuvant treatment after primary brain metastasis resection, *n* (%)	714			<.001	<0.001
Best supportive care		24 (38)	109 (17)		
Radiation therapy		12 (19)	158 (24)		
Radiation therapy and chemotherapy		6 (9.4)	176 (27)		
Radiation therapy and immunotherapy		20 (31)	143 (22)		
Radiation therapy and targeted therapy		2 (3.1)	64 (9.8)		
Pre‐operative antithrombotic intake, *n* (%)	880	23 (30)	187 (23)	.20	0.29
Ki67 status, *n* (%)	756			.034	0.061
<30%		44 (67)	366 (53)		
≥30%		22 (33)	324 (47)		
Survival, median (IQR)	878	3 (1–10)	9 (3–24)	<.001	<0.001
icPFS, median (IQR)	798	2 (1–6)	6 (2–13)	<.001	<0.001
ecPFS, median (IQR)	880	0 (0–2)	5 (1–12)	<.001	<0.001

*Note*: This table compares patients with non‐hemorrhagic brain metastases and hemorrhagic brain metastases to those with brain metastasis‐associated ICH. It includes detailed data on patient demographics, tumor characteristics, treatment details, and survival outcomes. Statistical testing (*p*‐values and corrected *q*‐values) is used to determine the significance of differences in these characteristics, shedding light on factors associated with ICH.

^a^ Wilcoxon rank sum test; Pearson's Chi‐squared test; Fisher's exact test.

^b^ False discovery rate correction for multiple testing.

Per institutional protocol, all patients with documented pre‐operative antithrombotic intake were considered actively exposed to these agents prior to surgery. In elective cases involving antiplatelet therapy, treatment was paused ≥7 days before surgery whenever feasible. In cases requiring urgent surgical intervention despite recent antiplatelet use, two platelet concentrates were transfused perioperatively to mitigate bleeding risk. For anticoagulants, a cessation window of at least 72 h was required before resection.

Outcome measures included overall survival (OS) and progression‐free survival (PFS) from the time of neurosurgical resection until the last follow‐up or death, analyzed using Kaplan–Meier and Cox proportional hazards models, with follow‐up periods estimated using the reverse Kaplan–Meier method like other studies in the field.^7^, ^19^, ^20^ Tumor progression was assessed retrospectively using RECIST 1.1 and iRANO criteria, with PFS divided into intracranial PFS (icPFS) and extracranial PFS (ecPFS).

### 2.5 Neuropathological data

PD‐L1 expression was evaluated using the PD‐L1 IHC 22C3 pharmDx assay (Dako‐Agilent) or a validated 22C3 protocol on the Dako‐Agilent Omnis platform. Tumor proportion score (TPS) was recorded in accordance with established scoring guidelines. For Ki‐67, immunohistochemical staining was performed on a Benchmark XT autostainer (Ventana Medical Systems) using standard antigen retrieval protocols (CC1 buffer, pH 8.0) and the monoclonal mouse anti‐MIB1 antibody (clone M7240, Dako; dilution 1:100).

### 2.6 Statistical analysis

All statistical analyses were performed using R Studio (v2023.09.0 + 463, The R Foundation for Statistical Computing, Boston, USA). Descriptive statistics summarized clinical and radiological data, with continuous variables compared using the Mann–Whitney *U* test and categorical variables using Fisher's exact or Chi‐squared tests. Logistic regression and Cox proportional hazards models assessed predictors of hemorrhage and overall survival (OS), adjusting for clinical covariates such as tumor volume, brain metastases, and extracranial disease. Hazard ratios (HR) with 95% confidence intervals (CI) were reported, with significance set at *p* < .05.

Generalized linear models predicted bleeding risk, focusing on two main models: Model 1 (Table 3A) assessed the risk of hemorrhage (hBrM or ICH‐BrM), while Model 2 specifically evaluated ICH‐BrM risk (Table 3B). The gtsummary R package (v0.4.3) was utilized to create the clinical data table. Model fit was evaluated using deviance and AIC criteria. Kaplan–Meier analysis estimated median progression‐free survival (PFS) and overall OS with 95% confidence intervals (CIs), with differences assessed via log‐rank tests and visualized using the survminer package (v0.4.9). Multivariable Cox regression for OS, icPFS, and ecPFS included cases with complete data and relevant clinical covariates. Additional analyses employed the dplyr (v1.1.4) and tidyverse (v2.0.0) R packages.

## 3 RESULTS

### 3.1 Comparison of non‐hemorrhagic brain metastasis, hemorrhagic brain metastasis and brain metastasis with ICH

Patient selection is displayed in the CONSORT diagram (Figure 1A). The study included 880 patients who underwent brain metastasis resection: 560 (64%) with non‐hBrM, 243 (30%) with hBrM, and 77 (9%) with ICH‐BrM. Median overall survival (OS) was 10.4 months [95% CI: 8.8–11.4], with icPFS at 6.1 months [95% CI: 5.3–6.9] and ecPFS at 5.9 months [95% CI: 5.2–6.8] (Figure S2A–L). The median follow‐up was 61.3 months [95% CI: 53.8–70.4].

The three groups are compared in Table 1A, whereas non‐hBrM patients (560, 64%) were also compared with patients with hBrM and ICH‐BrM (320 patients, 36%) (Table 1B), and non‐hBrM and hBrM (803 patients, 91%) were compared to ICH‐BrM (77 patients, 9%) (Table 1C). ICH‐BrM patients are described in detail in Table 2.

**TABLE 2:** Characteristics of patients presenting with ICH.

Variable	*N*	*N* = 77
Age, median (IQR)	58	63 (56–73)
Sex, *n* (%)	77	
Female		40 (52)
Male		37 (48)
KPS group, *n* (%)	40	
<80%		23 (58)
80–100%		17 (43)
GPA group, *n* (%)	34	
>2		9 (26)
0–2		25 (74)
Extracranial metastasis at baseline, *n* (%)	39	28 (72)
Underlying tumor entity, *n* (%)	77	
Breast cancer		5 (6.5)
Colorectal carcinoma		5 (6.5)
Esophageal carcinoma		1 (1.3)
Hepatocellular carcinoma (HCC)		2 (2.6)
Leiomyosarcoma		1 (1.3)
Melanoma		30 (39)
Non‐seminomatous germ cell tumor		1 (1.3)
Non‐small cell lung cancer (NSCLC)		17 (22)
Prostate cancer		3 (3.9)
Renal cell carcinoma (RCC)		5 (6.5)
Sarcoma		2 (2.6)
Small cell lung cancer (SCLC)		3 (3.9)
Seminoma		1 (1.3)
Thyroid carcinoma		1 (1.3)
Tumor volume (cm^3^), median (IQR)	36	21 (13–34)
Edema volume (cm^3^), median (IQR)	34	73 (34–109)
Anatomical site, *n* (%)	77	
Cerebellar		18 (23)
Other		59 (77)
Leading symptom, *n* (%)	77	
Alterations in behavior, disorientation, aphasia		13 (17)
Cerebellar symptoms		10 (13)
Classic HNV (headache, nausea, vomiting)		3 (3.9)
During staging		5 (6.5)
Headache		5 (6.5)
Incidental		1 (1.3)
Seizures		5 (6.5)
Sensory–motor symptoms or hemiparesis		22 (29)
Unknown		2 (2.6)
Vertigo and dyscoordination		5 (6.5)
Visual impairment		6 (7.8)
Hydrocephalus at baseline, *n* (%)	46	
Hydrocephalus		5 (11)
No hydrocephalus		41 (89)
Bleeding group, *n* (%)	77	
Hemorrhagic brain metastasis		0 (0)
Massive ICH		77 (100)
No hemorrhage		0 (0)
Total number categorized, *n* (%)	75	
≥2 or more brain metastases at baseline		30 (40)
1 brain metastasis at baseline		26 (35)
2 brain metastases at baseline		19 (25)
Absolute number, *n* (%)	69	
1		34 (49)
2		10 (14)
3		8 (12)
4		7 (10)
5		5 (7.2)
6		1 (1.4)
7		2 (2.9)
8		1 (1.4)
23		1 (1.4)
Localization, *n* (%)	74	
Both		18 (24)
Infratentorial		11 (15)
Supratentorial		45 (61)
Pre‐treatment status before primary brain metastasis resection, *n* (%)	67	
Pre‐treated before brain metastasis resection		40 (60)
Treatment‐naive		27 (40)
Adjuvant treatment after primary brain metastasis resection, *n* (%)	64	
Best supportive care		24 (38)
Radiation therapy		12 (19)
Radiation therapy and chemotherapy		6 (9.4)
Radiation therapy and immunotherapy		20 (31)
Radiation therapy and targeted therapy		2 (3.1)
Pre‐operative anticoagulation or blood thinner intake, *n* (%)	77	23 (30)
Ki67 status, *n* (%)	66	
<30%		44 (67)
≥30%		22 (33)
Survival, median (IQR)	77	3 (1–10)
icPFS, median (IQR)	77	0.9 (0.0–2.5)
ecPFS, median (IQR)	77	0.6 (0.0–2.6)

*Note*: This table presents the clinical characteristics of 77 patients who showed ICH in their pre‐operative imaging before brain metastasis resection. The table includes demographic details such as the median age of the patients (63 years, IQR 56–73), and their gender distribution, with 52% female and 48% male. The majority of patients (72%) had extracranial metastasis at baseline, and melanoma was the most common underlying tumor type (39%), followed by non‐small cell lung cancer (22%). Anatomically, 23% of the tumors were located in the cerebellum, and 29% of patients presented with sensory‐motor symptoms or hemiparesis as the leading symptom. Pre‐operative antithrombotic drug use was reported in 30% of patients, and 33% had a Ki67 proliferation index of 30% or higher. In addition, adjuvant treatments post‐resection included best supportive care (38%) and combinations of radiation therapy with chemotherapy, immunotherapy, or targeted therapy.

**TABLE 3A:** Logistic regression model summary (event = bleeding [hemorrhage + ICH]).

Predictor	Estimate	Std. error	*z* value	Pr(>|*z*|)	Significance
(Intercept)	−2.6088	0.4911	−5.3120	0.0000	***
Pre‐treatment status before primary brain metastasis resection (treatment‐naive)	−0.1836	0.1928	−0.9520	0.3411	
Pre‐operative antithrombotic intake (yes)	−0.0790	0.2088	−0.3780	0.7051	
Entity (melanoma)	2.9685	0.4928	6.0240	0.0000	***
Entity (NSCLC)	1.5764	0.4641	3.3970	0.0007	***
Entity (Other)	1.8534	0.4844	3.8260	0.0001	***
Tumor volume group (≥ 11.4)	0.4331	0.1809	2.3950	0.0166	*
Total number categorized (1 brain metastasis)	−0.0750	0.2288	−0.3280	0.7431	
Total number categorized (2 brain metastases)	−0.4020	0.2237	−1.7970	0.0723	.
Extracranial metastases at baseline (yes)	0.2905	0.1908	1.5230	0.1278	

*Note*: This table presents a logistic regression model assessing predictors for bleeding events (combining patients with either signs of intralesional hemorrhage or intracranial hemorrhage [ICH]). The predictor variables include pre‐treatment status, pre‐operative antithrombotic intake, tumor type (Melanoma, NSCLC, other), tumor volume group (categorized as ≥11.4), and other clinical factors such as total brain metastasis classification and extracranial metastasis. Estimates, standard errors, *z*‐values, and *p*‐values are provided for each variable to determine their significance in predicting bleeding events. Significant predictors include tumor type and tumor volume group, with the strongest associations observed for melanoma and NSCLC tumors.

**TABLE 3B:** Logistic regression model summary (event = no bleeding and hemorrhage vs. ICH).

Predictor	Estimate	Std. error	*z* value	Pr(>|*z*|)	Significance
(Intercept)	−20.7401	1264.5348	−0.0160	0.9869	
Pre‐treatment status before primary brain metastasis resection (treatment‐naive)	−0.7005	0.4042	−1.7330	0.0831	.
Pre‐operative antithrombotic intake (yes)	0.6599	0.4216	1.5650	0.1175	
Entity (melanoma)	18.2168	1264.5347	0.0140	0.9885	
Entity (NSCLC)	16.4555	1264.5347	0.0130	0.9896	
Entity (other)	16.9486	1264.5347	0.0130	0.9893	
Tumor volume group (≥11.4)	1.2471	0.4521	2.7590	0.0058	**
Total number categorized (1 brain metastasis)	0.3294	0.5354	0.6150	0.5384	
Total number categorized (2 brain metastases)	−0.3785	0.4730	−0.8000	0.4236	
Extracranial metastases at baseline (yes)	0.2164	0.4564	0.4740	0.6355	

*Note*: This table summarizes the results of a logistic regression model focusing on differentiating between no bleeding or hemorrhage versus ICH. Variables analyzed include pre‐treatment status, pre‐operative antithrombotic use, tumor type, tumor volume, and other clinical characteristics. Although most variables show no significant association with ICH, tumor volume group (≥11.4) was a significant predictor (*p* = .0058), suggesting that larger tumor volume is a key risk factor for ICH. Estimates, standard errors, *z*‐values, and *p*‐values are displayed to provide insights into the factors influencing the likelihood of ICH.

ICH‐BrM was associated with larger tumor volumes (median: 21 mL; IQR: 13–34) compared to hBrM (14 mL; IQR: 6–28) and non‐hBrM (12 mL; IQR: 6–21) (*p*‐adjusted = .016) (Table 1C). The total number of lesions was notably different between groups: 30 patients with ICH‐BrM (40%) and 94 patients presenting with hBrM (30%) had ≥2 brain metastatic lesions at baseline, whereas 151 (27%) non‐hBrM patients had ≥2 brain metastatic lesions at baseline (*p*_adjust_ = .016) (Table 1A). Symptoms varied, with sensory‐motor deficits or hemiparesis being most frequent in 29% in ICH‐BrM compared to 19% in patients without ICH. Extracranial metastases were more frequent in the ICH‐BrM group (72%) compared to hBrM (56%) and non‐hBrM (44%) (*p*_adjust_ < .001) (Table 1A). Tumor location varied slightly, with the frontal lobe as the most common site in 32% of cases with and without ICH and parietal lobe involvement more common in ICH‐BrM (25%) than non‐hBrM (17%) (Tables 1A, 1B, 1C). As to the pre‐treatment of brain metastases before primary resection including antithrombotic medication, there was no significant difference between the three groups. Yet, ICH‐BrM were more likely to receive best supportive care and less likely to receive adjuvant radiotherapy or adjuvant radiation therapy with systemic treatment (Table 1A). Lastly, patients with ICH had a lower median Ki‐67 index but higher PD‐L1 tumor proportion score within the resected brain metastasis tissue (Table 1A and Figure S4A,B).

### 3.2 Impact of hemorrhage or ICH on survival and functional outcome measures

In total, 679 patients died and 201 were still alive on the last day of follow‐up. Kaplan–Meier analysis showed significantly shorter OS for ICH‐BrM (3.8 months; 95% CI: 2.4–10.4) compared to hBrM (8.1 months; 95% CI: 6.0–10.6) and non‐hBrM (11.8 months; 95% CI: 10.5–13.6). However, multivariable analysis indicated that hemorrhage type did not independently predict OS. Instead, factors such as age (≥65 years), extracranial metastases, and tumor volume (≥11.4 cm^3^) were associated with higher mortality risk (HR = 1.53–1.77, *p* < 0.001). Single brain metastasis and adjuvant therapies were linked to improved survival (HR = 0.62, *p* = .002). Non‐hBrM had a median icPFS of 7.5 months [95% CI: 6.4–8.4], hBrM had a median icPFS of 5.1 months [95% CI: 4.3–7.2], and ICH‐BrM had icPFS of 2.7 months [95% CI: 1.9–7.4]. The ICH‐BrM group had a median ecPFS of 3.3 months [95% CI: 1.9–7.4], while those without ICH had a median ecPFS of 6.6 months [95% CI: 5.8–7.8].

Patients with ICH‐BrM also had poorer functional outcomes, with 58% having a post‐operative Karnofsky performance score (KPS) below 80%, compared to 44% in hBrM and 38% in non‐hBrM groups (*p*_adjust_ = .045). (Tables 1A, 1B, 1C). Mortality rates within 30 days of surgery were highest in the ICH‐BrM group (22.1%), followed by hBrM (11.1%) and non‐hBrM (3.57%) (*p* < .001) (Figure S3).

**FIGURE 2:**
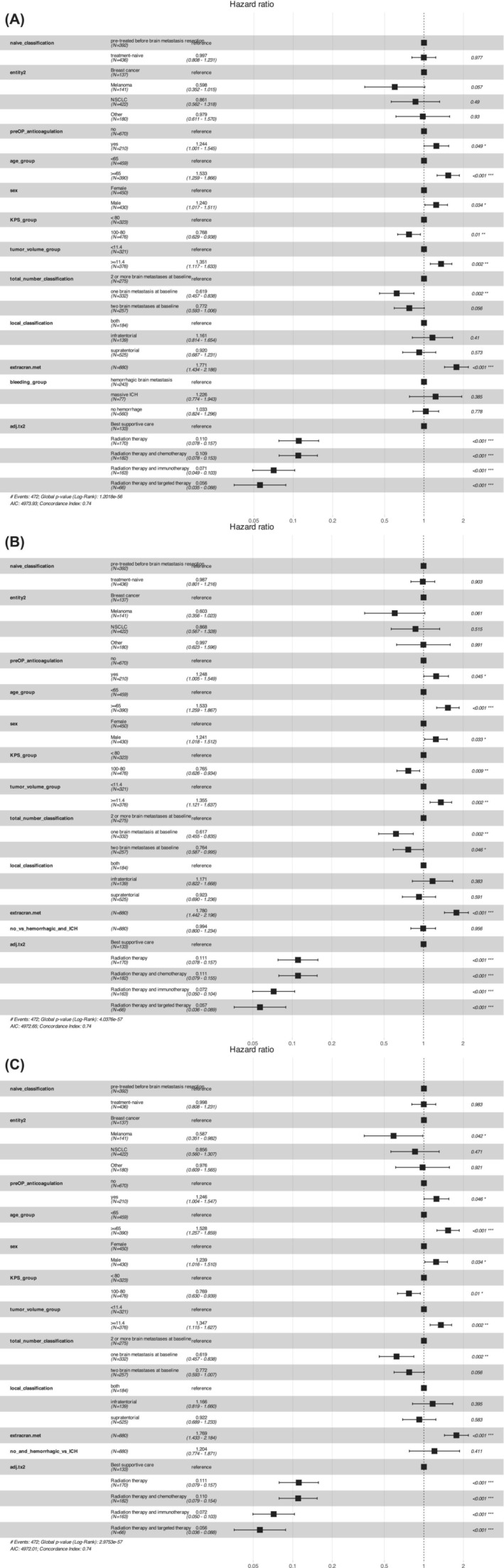
(A) Cox proportional hazards model for OS—non‐hemorrhagic vs. hemorrhagic vs. ICH. This figure presents the results of a Cox proportional hazards model, with patients being stratified into three groups: non‐hemorrhagic brain metastases, brain metastases with signs of intralesional hemorrhage and brain metastases associated with ICH, evaluating the impact of various clinical and treatment‐related variables on overall survival (OS) in patients with brain metastasis. Hazard ratios (HRs) and corresponding 95% confidence intervals (CIs) are shown for each variable, with significant predictors of OS highlighted. The model includes the factors age, tumor volume, extracranial metastasis, pre‐operative antithrombotics, bleeding group, and post‐operative treatment modalities (e.g., radiation and systemic therapies). (B) Cox proportional hazards model for OS with bleeding group (no hemorrhage vs. hemorrhagic and ICH). This hazard plot visualizes the results of a Cox proportional hazards model evaluating the impact of clinical and treatment‐related factors on overall survival (OS) in patients stratified by bleeding status: no hemorrhage versus hemorrhagic brain metastasis and ICH. Similar key variables were included as in the first model or Panel A: age, tumor volume dichotomized, presence of extracranial metastasis, concurrent pre‐operative antithrombotics, and type of post‐operative treatment, including radiation therapy with or without systemic therapies. The hazard ratios (HRs) with 95% confidence intervals (CIs) are depicted for each factor, indicating significant predictors of survival outcomes. (C) Cox proportional hazards model for OS (non‐hemorrhagic and hemorrhagic metastases vs. ICH). This figure presents a Cox proportional hazards model examining the impact of various clinical factors and treatments on overall survival (OS) in patients stratified into two groups: (1) patients with no hemorrhage or hemorrhagic brain metastases and (2) patients with ICH, see also Panels A and B.

We evaluated the impact of pre‐operative concurrent antithrombotic medication (either anticoagulants or antiplatelets) and the impact of the different bleeding types (non‐hBrM vs. hBrM vs. ICH‐BrM or non‐hBrM vs. hBrM and ICH‐BrM or non‐hBrM and hBrM vs. ICH‐BrM) on survival, demonstrated that signs of hBrM or ICH‐BrM were not independently associated with survival in our cohort (Figure 2A–C). Across all three models, age ≥ 65 years (HR = 1.53, *p* < .001), extracranial metastasis (HR = 1.77–1.78, *p* < .001), and tumor volume ≥ 11.4 cm^3^ (HR = 1.35, *p* = .001–.002) were consistently associated with a significantly higher risk of death (Figure 2A–C). Notably, single brain metastasis at baseline was associated with a lower risk of death (HR = 0.62, *p* = .002). Furthermore, male sex (HR = 1.24, *p* = .03) and lower KPS (<80%) (HR = 0.76–0.77, *p* < .01) were also significant factors, indicating worse survival in males and better survival in patients with higher functional status (Figure 2A–C). Patients who had received adjuvant therapies, that is, radiation with or without chemotherapy, immunotherapy (with checkpoint inhibitors) or targeted therapies demonstrated significantly improved survival outcomes across all models (*p* < .001) (Figure 2A–C).

### 3.3 Predictors of hemorrhage or ICH

Melanoma, NSCLC or other entities (reference: breast cancer) and tumor volume were independent predictors for the occurrence of hemorrhage or ICH, whereas only tumor volume was predictive of the occurrence of ICH (Table 2).

### 3.4 Symptom profiles and clinical relevance of massive ICH

We investigated the clinical symptom profiles of patients with hemorrhagic brain metastases, massive ICH, and those without hemorrhage. Among the grouped binary symptoms analyzed, focal neurological deficits (FND), which included hemiparesis (motor deficit), sensory deficit, aphasia, visual impairment, vertigo or cerebellar signs, were significantly associated with hemorrhage status (Chi^2^ = 11.87, *p* = .0026). FND symptoms were most prevalent in the massive ICH group (53.3%), followed by the hemorrhagic brain metastasis group (43.5%), and least frequent in patients without hemorrhage (35.1%). Other binary symptoms, including increased intracranial pressure (ICP)—headache, nausea, or vomiting—seizures, altered mental status (AMS), and incidental symptoms, did not exhibit statistically significant differences between groups (Figure S5A). Examination of the full spectrum of leading symptoms at diagnosis revealed a significant difference in symptom distribution across the three groups (Chi^2^ = 43.03, *p* = .0047). Although the difference in none of the individual symptoms remained significant after false discovery rate correction at a strict threshold of *q* < 0.05, two symptoms, sensory–motor deficits, and vertigo/cerebellar symptoms, bordered on significance (*q* ≈ 0.056), suggesting potential group differences in symptom presentation that warrant further study (Figure S5B).

Finally, we analyzed the specific clinical relevance of the hemorrhagic incident in our massive ICH patients by reviewing patient charts and symptom reports at the time of hemorrhage before resection. We were able to reconstruct the timeline of appearance of clinical symptoms and diagnosis of ICH in metastases for 69 of our 77 massive ICH patients (90%). Of these, in 62 patients (90%), the hemorrhage was detected shortly after symptomatic deterioration or acute onset of symptoms and required emergency treatment. In seven patients (10%), the hemorrhage did not cause any clinical symptoms or did not cause a worsening of symptoms already present. The two most frequent leading symptoms were observed in more than half of the patients, with sensory–motor symptoms or hemiparesis in 22 patients (32%) and alterations of behavior or disorientation in 14 patients (20%). Thus, hemorrhage, especially with larger volume can be clinically relevant causing symptoms and often resulting in necessary urgent treatment.

The differing symptom profiles between groups emphasize the importance of careful neurological assessment, as symptom patterns may aid in detecting hemorrhagic complications in brain metastases. Future studies should further clarify these relationships and their impact on clinical management.

## 4 DISCUSSION

Our study identified larger tumor size and melanoma as significant predictors of hBrM and ICH‐BrM, consistent with prior research. However, neither hBrM nor ICH‐BrM independently predicted OS, emphasizing the dominance of tumor burden, extracranial metastases, and adjuvant treatments in driving outcomes. This analysis builds on previous work by including a brain metastasis cohort unmatched in size compared to most prior studies,^4^, ^5^, ^6^, ^7^, ^19^ allowing for robust multivariable modeling. Similar to recently published work by Rauschenbach et al. (including 54 patients with ICH, total cohort size: 229 patients) and Hamed et al. (122 patients with pre‐operative hemorrhagic transformation, total cohort size: 357), we exclusively focused on patients with conclusive histopathological classification following neurosurgical resection with evidence of tumor cells within resected (hemorrhagic) brain metastasis tissue.^7^, ^19^

While earlier studies suggested hemorrhagic transformation of brain metastases as a survival predictor (Rauschenbach et al. and Hamed et al. Cox showing proportional hazard models with HR: 1.53; 95% CI = 1.04–2.24; *p* = .03 and, HR: 1.4, 95% CI = 1.1–1.8, *p* = .009, respectively^7^, ^19^), our findings indicate that hemorrhage type alone does not substantially impact OS when adjusted for clinical and radiological covariates. Instead, survival outcomes are influenced by factors such as age, functional status (KPS), and treatment modality, underscoring the importance of comprehensive management strategies.

The discrepancies between the studies may be linked to certain cohort composition, sample size, and methodological differences like differences in inclusion criteria or definitions of hemorrhage. Our institution as the largest neurosurgical clinic in Germany might have included more complex cases and only a certain selection of brain metastases cases. Because hemorrhage may interact with other variables, such as tumor volume, functional status, or timing of surgery, to influence survival indirectly, the cohort definition and selection of patients treated at a center conducting a study, as well as the clinical management of examinations and treatments of patients might have influenced the results. Of course, certain statistical adjustments can also always impact reported results.

Notably, melanoma's association with ICH‐BrM aligns with its known vascular propensity.^7^, ^19^ Non‐small cell lung cancer (NSCLC) and other non‐breast cancer entities also emerged as contributing risk factors. These findings are consistent with those from prior studies, which also reported elevated risks of hemorrhage in patients with specific tumor types.^5^, ^7^, ^19^ Similarly, tumor volume emerged as a critical risk factor, highlighting the need for vigilant monitoring and tailored surgical planning in patients with larger metastases. In contrast, pre‐operative antithrombotic use did not increase hemorrhagic risk, supporting its cautious continuation in select cases. These findings contribute to refining peri‐operative risk stratification and management. Our results underscore the importance of adjuvant therapies, particularly combinations involving radiation and systemic treatments, in improving survival.

Future studies should prioritize prospective, multi‐institutional designs to validate these findings and explore targeted interventions for high‐risk patients. A focus on prospective studies with uniform hemorrhage definitions, balanced covariates, and sufficiently powered subgroups could enable determining whether hemorrhage itself confers independent prognostic relevance or reflects surrogate markers of disease severity.

### 4.1 Limitations

Our study has several limitations. Restricting the cohort to resected brain metastases may limit generalizability, as these patients differ from those with unresectable or non‐operable lesions. Additionally, the single‐center design may introduce selection bias, as our institution is the largest neurosurgical clinic in Germany and regularly manages complex cases. While grouping pre‐operative antithrombotic medications (including oral anticoagulants, antiplatelets and low‐molecular‐weight heparins) avoided small subgroups to maintain sufficient statistical power, it limited differentiation between drug‐specific effects on hemorrhage risk. This approach is similar to the work by Hamed et al. and Rauschenbach et al.^7^, ^19^, ^20^, ^21^, ^22^ A systematic review and meta‐analysis by Giustozzi et al. support our observations on no significant association between anticoagulation use and ICH‐BrM.

While our data did not show a significant association between pre‐operative antithrombotic therapy and hemorrhagic transformation, these findings should not be interpreted as evidence of safety in patients with hemorrhagic brain metastases. Selection bias may have influenced which patients received surgery or anticoagulation in the first place, potentially excluding those at highest bleeding risk. Moreover, we were unable to differentiate between drug classes (e.g., DOACs vs. antiplatelets), dosages, or specific indications due to limited subgroup sizes. Thus, our findings support the feasibility of antithrombotic use in selected cases but do not establish its overall safety. Prospective studies with standardized documentation are needed to clarify risk profiles across different treatment regimens.

Additionally, a limitation of our analysis is the use of a 30 mm ICH diameter cut‐off, which, while based on prior clinical, neurooncological and neuroimaging studies and reflecting its prior use in clinical scoring systems, remains somewhat arbitrary. Specifically, the ICH score^23^ includes a ≥30 mm volume cut‐off as a predictor of poor prognosis in spontaneous intracerebral hemorrhage. Furthermore, retrospective neurosurgical oncology studies and neuroimaging studies on outcome prediction after ICH have applied similar cut‐off values to define space‐occupying hematomas with relevant clinical impact: Haider et al. found a maximum coronal diameter of ~3.5 cm to indicate worse outcomes in patients with acute cerebral hemorrhage.^24^ Nag et al., in turn, describe a hematoma volume > 30 cm^3^ to be associated with early mortality in ICH.^25^ However, it must be acknowledged that the 30 mm ICH diameter cut‐off may not fully capture the clinical heterogeneity of hemorrhagic presentations.^26^, ^27^, ^28^

Further, the retrospective design and inclusion of patients over a 14‐year span could introduce variability due to evolving treatment protocols and imaging technologies. ICH‐BrM often must undergo surgical emergency evacuation without pre‐operative MRI, leading to reliance on CT imaging for hemorrhage assessment in some cases, which may affect measurement accuracy. Blooming effects on T2*‐weighted or SWI sequences could also overestimate hemorrhage size compared to CT.

Finally, the lack of covariate balancing across patient groups may introduce residual confounding, and the absence of detailed surgical complication data limits insights into post‐operative outcomes. Future multi‐institutional, prospective studies with standardized imaging and treatment protocols are needed to validate our findings and improve risk stratification.

## 5 CONCLUSION

This study provides valuable insights into the risk factors and outcomes associated with hemorrhagic brain metastases (hBrM) and intracranial hemorrhage‐associated brain metastases (ICH‐BrM). Larger tumor size and melanoma were identified as significant predictors of hemorrhage, though neither hBrM nor ICH‐BrM independently predicted overall survival. Instead, survival was driven by tumor burden, extracranial metastases, and adjuvant treatments.

These findings underscore the importance of comprehensive risk assessment and tailored management strategies for patients with brain metastases. Future research should focus on prospective, multi‐institutional studies to validate these results and refine patient management strategies.

## AUTHOR CONTRIBUTIONS

**Melisa S. Guelen:** Conceptualization; investigation; writing – original draft; methodology; visualization; writing – review and editing; formal analysis; project administration; data curation. **Kiarash Ferdowssian:** Conceptualization; investigation; data curation; writing – review and editing; formal analysis. **Niklas Jung:** Conceptualization; investigation; writing – review and editing; data curation; formal analysis. **Hava N. Celik:** Formal analysis; data curation; investigation. **Andrea Dell'Orco:** Writing – review and editing. **Semil Eminovic:** Writing – review and editing. **Anton Früh:** Writing – review and editing. **Majd Samman:** Writing – review and editing; supervision. **Güliz Acker:** Writing – review and editing. **Arend Koch:** Writing – review and editing. **Helena Radbruch:** Writing – review and editing. **Michael Scheel:** Writing – review and editing. **Mike P. Wattjes:** Writing – review and editing. **Julia Onken:** Writing – review and editing; supervision. **Peter Vajkoczy:** Writing – review and editing; supervision. **Nils Hecht:** Writing – review and editing. **Jawed Nawabi:** Conceptualization; investigation; data curation; writing – original draft; methodology; project administration; writing – review and editing; supervision; validation. **David Wasilewski:** Conceptualization; investigation; data curation; writing – original draft; methodology; formal analysis; software; project administration; writing – review and editing; supervision.

## FUNDING INFORMATION

This research was not supported by any specific grants.

## CONFLICT OF INTEREST STATEMENT

The authors declare no conflict of interest.

## ETHICS STATEMENT

Ethical approval was obtained (EA1/174/24) by the Ethics Subcommittee Members at Campus Charité Mitte (CCM) (Chair: Prof. Dr. med. R. Uebelhack) at Charité's Ethics Committee; the requirement for informed consent was waived by the Institutional Review Board, and the study is in accordance with the ethical standards outlined in the Declaration of Helsinki.

## Supporting information


**FIGURE S1:** (A, B) Nonarbitrary cut‐off determination for tumor volume and edema volume using maximally selected rank statistics for survival.
**FIGURE S2:** (A–C) Outcome of the total cohort. (D–F) Outcome compared between the three patient groups: non‐hemorrhagic BrM, hBrM and ICH‐BrM. (G–I) Outcome compared between the two patient groups: non‐hemorrhagic BrM vs. hBrM or ICH‐BrM. (J–L) Kaplan–Meier survival curves comparing patients with non‐hemorrhagic BrM and hBrM vs. ICH‐BrM.
**FIGURE S3:** Thirty‐day mortality rate stratified by the type of hemorrhage in patients with resected brain metastases.
**FIGURE S4:** (A, B) Distribution of PDL1 TPS and Ki67 in resected brain metastasis tissue across patient groups.
**FIGURE S5:** (A) Grouped symptoms by hemorrhagic status. (B) Heatmap of FDR‐corrected *p*‐values for individual symptom associations across hemorrhage groups.

## Data Availability

The R code is available on Github: https://github.com/dasilew/clinicalNSCLC_1. Anonymized raw data will be made available from the corresponding author upon reasonable request.
