# Supplementary material for: Outcomes and risk factors of hemorrhage in patients with resected brain metastases
Source: Int J Cancer. 2025 Dec 7;158(8):2120–38. doi: 10.1002/ijc.70250 (PMC12922642; doi:10.1002/ijc.70250)
Supplement: Supplementary file 1 — FIGURE S1: (A, B) Nonarbitrary cut‐off determination for tumor volume and edema volume using maximally selected rank statistics for survival. FIGURE S2: (A–C) Outcome of the total cohort. (D–F) Outcome compared between the three patient groups: non‐hemorrhagic BrM, hBrM and ICH‐BrM. (G–I) Outcome compared between the two patient groups: non‐hemorrhagic BrM vs. hBrM or ICH‐BrM. (J–L) Kaplan–Meier survival curves comparing patients with non‐hemorrhagic BrM and hBrM vs. ICH‐BrM. FIGURE S3: Thirty‐day mortality rate stratified by the type of hemorrhage in patients with resected brain metastases. FIGURE S4: (A, B) Distribution of PDL1 TPS and Ki67 in resected brain metastasis tissue across patient groups. FIGURE S5: (A) Grouped symptoms by hemorrhagic status. (B) Heatmap of FDR‐corrected p‐values for individual symptom associations across hemorrhage groups. [file IJC-158-2120-s001.pdf]

Supplementary material

Outcomes and Risk Factors of Hemorrhage in Brain Metastases

Melisa S. Guelen, Kiarash Ferdowssian, Niklas Jung, Hava N. Celik, Andrea Dell'Orco, Semil Eminovic, Anton Früh, Majd Samman, Güliz Acker, Arend Koch, Helena Radbruch, Michael Scheel, Mike P. Wattjes, Julia Onken, Peter Vajkoczy, Nils Hecht, Jawed Nawabi, David Wasilewski

Table of Contents

Supplementary figure 1A-B	Nonarbitrary cut-off determination for tumor volume and edema volume
Supplementary figure 2A-C	Outcome of the total cohort
Supplementary figure 2D-F	Outcome compared between the three patient groups: non-hemorrhagic BrM, hBrM and ICH-BrM
Supplementary figure 2G-I:	Outcome compared between the two patient groups: non-hemorrhagic BrM vs. hBrM or ICH-BrM
Supplementary figure 2J-L	Kaplan-Meier survival curves comparing patients with non-hemorrhagic BrM and hBrM vs. ICH-BrM
Supplementary figure 3	30-day mortality rate stratified by the type of hemorrhage in patients with resected brain metastases
Supplementary figure 4A-B:	Distribution of PDL1 TPS and Ki67 in resected brain metastasis tissue across patient groups)
Supplementary figure 5A	Grouped Syptoms by Hemorrhagic Status
Supplementary figure 5B	Heatmap of FDR-corrected p-values for individual symptom associations across hemorrhage groups

1A

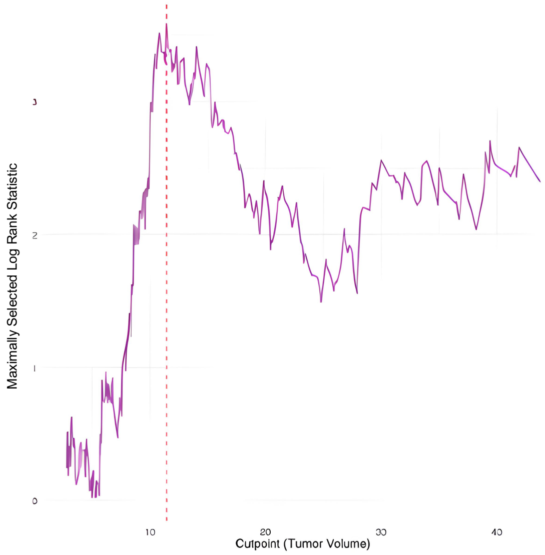

1B

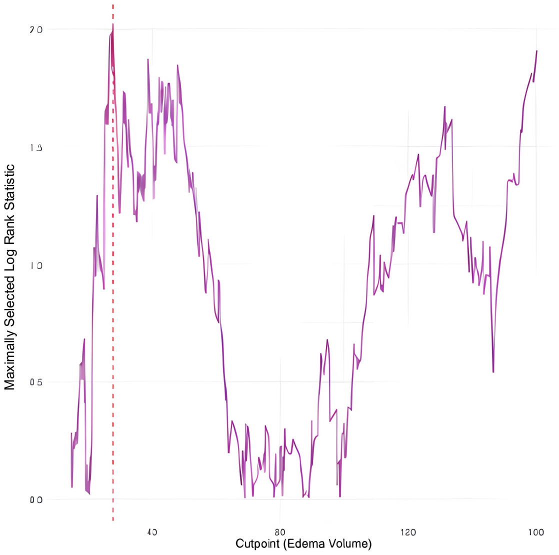

Supplementary figure 1A-B: Nonarbitrary cut-off determination for tumor volume and edema volume using maximally selected rank statistics for survival:

The maximally selected log-rank statistic for tumor volume is displayed as a function of various tumor volume cut-points. The vertical red dashed line indicates the optimal cut-point of 11.4 cm^3, which was selected to maximize the survival difference based on tumor volume (**A**). The maximally selected log-rank statistic for edema volume is displayed as a function of various edema volume cut-points with the red dashed line indicating the optimal cut-point of 27.8 cm^3, which was selected to maximize the survival difference based on edema volume (**B**).

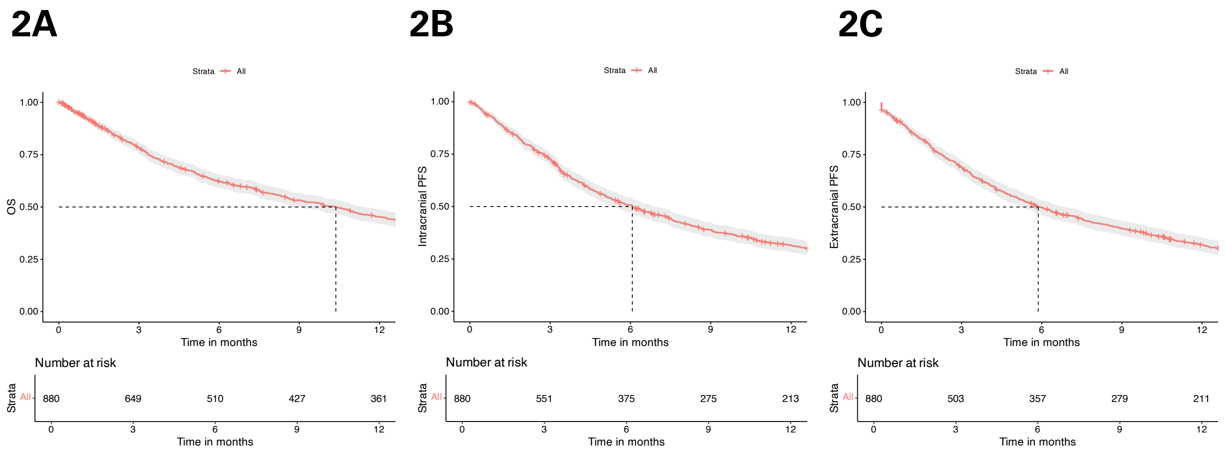

Supplementary figure 2A-C: Outcome of the total cohort:

Kaplan-Meier survival curves for overall survival (OS) of the total cohort, 10.4 months [95% CI: 8.8 – 11.4] (1A), for intracranial progression-free survival (icPFS) (6.1 months [95%CI: 5.3 – 6.9]) (1B), extracranial progression-free survival (ecPFS) was 5.9 months [95%CI: 5.2 – 6.8] (1C).

2D 2E 2F

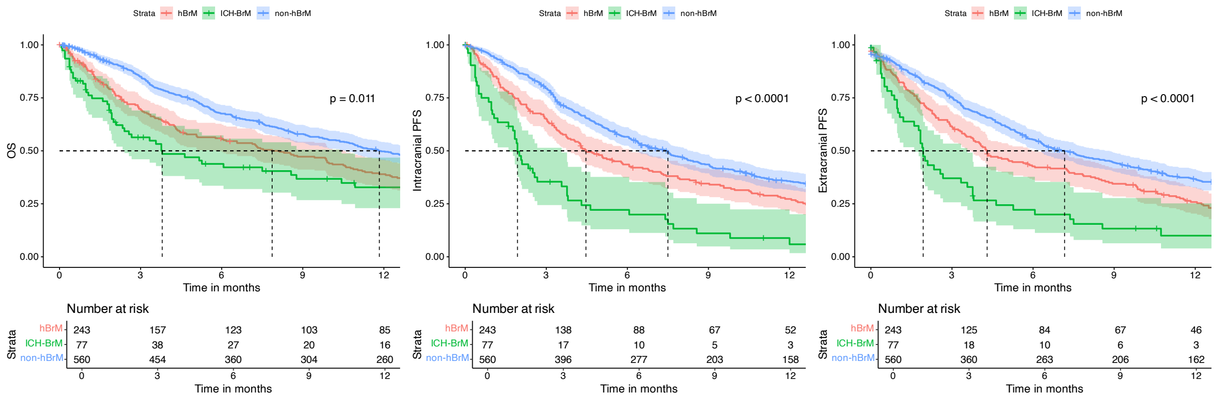

Supplementary figure 2D-F: Outcome compared between the three patient groups: non-hemorrhagic BrM, hBrM and ICH-BrM:

Patients with non-hemorrhagic BrM had a median OS of 11.83 months (95% CI: 10.63–13.6), patients with hBrM had a median OS of 7.87 months (95% CI: 5.77–10.6), and patients with ICH-BrM had a median OS of 3.80 months (95% CI: 2.40–10.4) (**D**). Median icPFS: non-hBrM (n = 560), hBrM group (n = 243), and ICH-BrM group (n = 77). The icPFS for patients with no hemorrhage was 7.50 months (95% CI: 6.37–8.33), whereas patients with hBrM had a median icPFS of 4.47 months (95% CI: 3.77–6.07). Patients with ICH-BrM had the shortest icPFS, with a median of 1.93 months (95% CI: 1.60–3.57) (**E**). Median ecPFS: Patients without hemorrhage had a median ecPFS of 7.50 months (95% CI: 6.37–8.33), patients with hBrM had a median ecPFS of 4.30 months (95% CI: 3.70–6.07), and patients with ICH-BrM had a median ecPFS of 1.93 months (95% CI: 1.60–3.57) (**F**).

2G 2H 2I

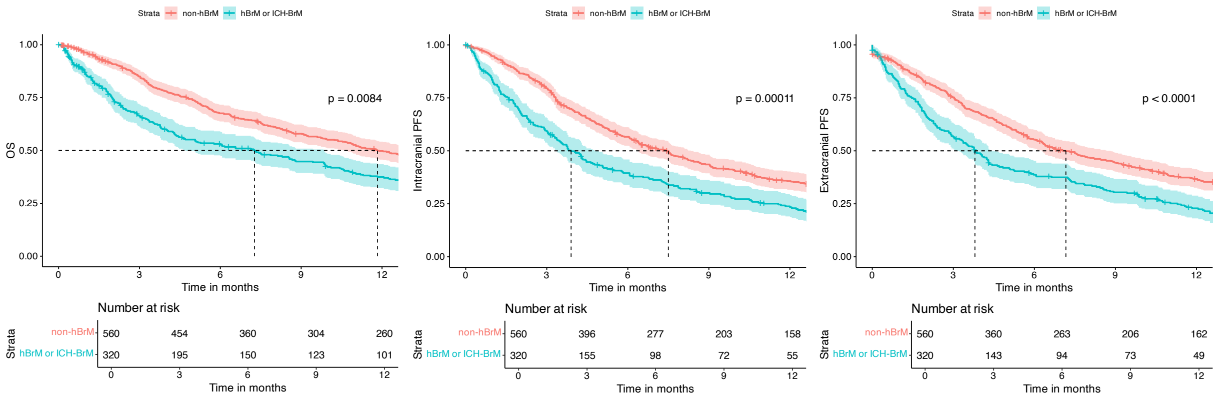

Supplementary figure 2G-I: Outcome compared between the two patient groups: non-hemorrhagic BrM vs. hBrM or ICH-BrM:

Kaplan-Meier survival curves comparing patients with non-hemorrhagic BrM, with hBrM or ICH-BrM in the context of brain metastasis resection. The analysis was conducted for OS, icPFS, and ecPFS. For OS (**G**), patients non-hemorrhagic BrM (n = 560) had an OS of 11.83 months (95% CI: 10.6-13.57), while those with hBrM or ICH-BrM (n = 320) had a median survival of 7.27 months (95% CI: 4.7-9.87). For icPFS (**H**), patients without hemorrhage had a median icPFS of 7.5 months (95% CI: 6.37-8.33), compared to 3.9 months (95% CI: 3.37-4.77) in patients with hBrM or ICH-BrM. For ecPFS (**I**), patients with non-hemorrhagic BrM had a median ecPFS of 7.17 months (95% CI: 6.27-8.83), whereas those with hBrM or ICH-BrM had a median ecPFS of 3.80 months (95% CI: 3.07-4.43).

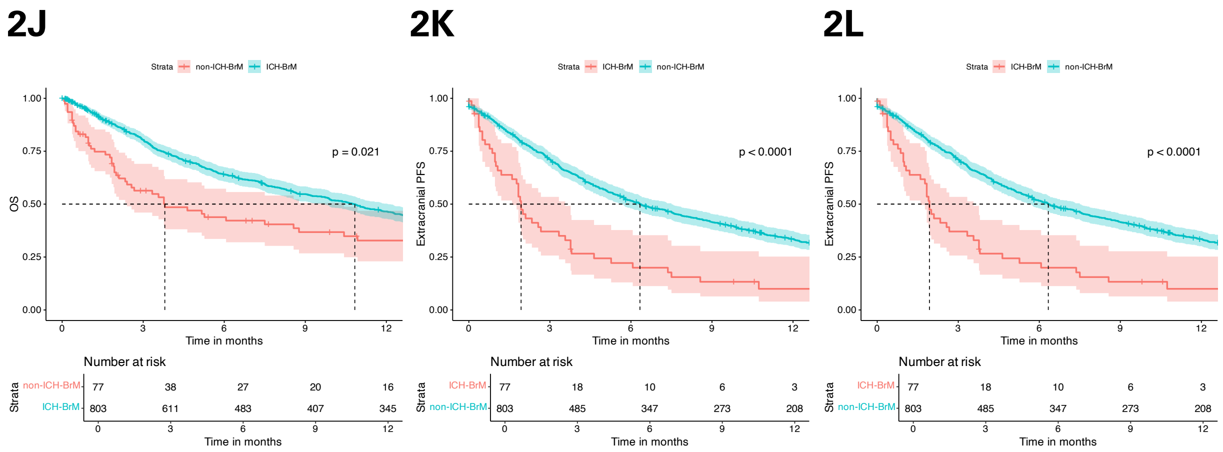

Supplementary figure 2J-L: Kaplan-Meier survival curves comparing patients with non-hemorrhagic BrM and hBrM vs. ICH-BrM:

The analysis was conducted for OS, icPFS, and ecPFS. For OS (**J**), patients with ICH-BrM (n = 77) had an OS of 3.8 months (95% CI: 2.4-10.4), while patients without BrM-ICH (n = 803) had a significantly better OS of 10.8 months (95% CI: 9.6-12.1). For icPFS (**K**), patients with massive ICH had a median icPFS of 1.93 months (95% CI: 1.60-3.57), compared to median icPFS of 6.43 months (95% CI: 5.77-7.50) in patients without ICH-BrM. For ecPFS (**L**), patients with ICH-BrM had a median ecPFS of 1.93 months (95% CI: 1.60-3.57), whereas those without ICH-BrM had a median ecPFS of 6.33 months (95% CI: 5.63-7.50).

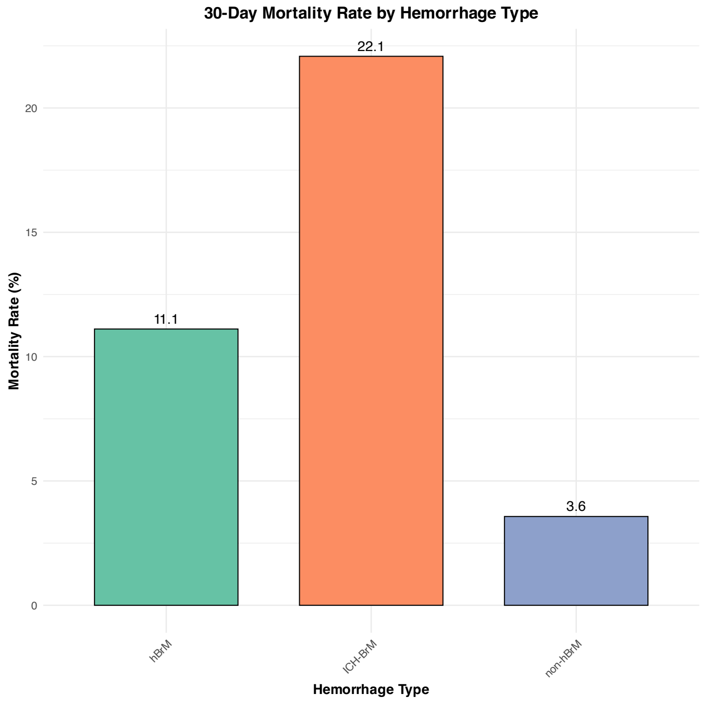

Supplementary figure 3: 30-day mortality rate stratified by the type of hemorrhage in patients with resected brain metastases:

This bar plot illustrates the 30-day mortality rates across three patient groups: non-hemorrhagic BrM (n=560), hBrM (n=243), and ICH-BrM (n=77). The 30-day mortality rate was highest in the massive ICH group at 22.1%, followed by the hBrM group at 11.1%, and was lowest in the non-hemorrhagic BrM group at 3.57%. The bars represent the percentage of deaths within 30 days for each group, with the exact mortality rates displayed on top of each bar. A Chi-square test of independence confirmed a statistically significant difference in mortality rates between the groups ($\chi^2 = 41.71$, df = 2, $p < 0.001$), indicating that patients with ICH-BrM had a substantially higher 30-day mortality rate compared to the other groups.

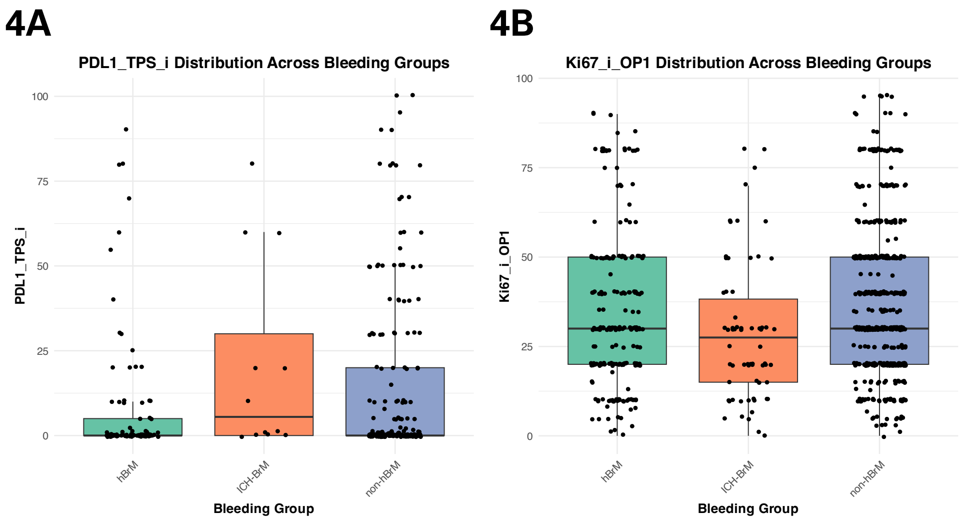

Supplementary figure 4A-B: Distribution of PDL1 TPS and Ki67 in resected brain metastasis tissue across patient groups):

This box plot illustrates the distribution of PDL1_TPS_i across three distinct patient groups: non-hemorrhagic brain metastases, hBrM, and ICH-BrM showing the central tendency and spread of the PDL1_TPS_i values (jittered points) overlaid (**A**). This figure depicts the distribution of Ki67_i_OP1 across the same three patient groups. The ICH-BrM group has a broader range of Ki67_i_OP1 values, while the other two groups showed different levels of spread (**B**).

The ANOVA results for PDL1_TPS_i showed no statistically significant differences between the bleeding groups (non-hBrM, hBrM, and ICH-BrM; p = 0.093), indicating similar distribution across groups. However, for Ki67_i_OP1, the ANOVA revealed significant differences between groups (p = 0.0046). Post-hoc analysis for Ki67_i_OP1 indicated that the difference between non-hBrM and ICH-BrM was significant (p = 0.0066), with ICH-BrM having lower values, while the other pairwise comparisons were not statistically significant.

5A

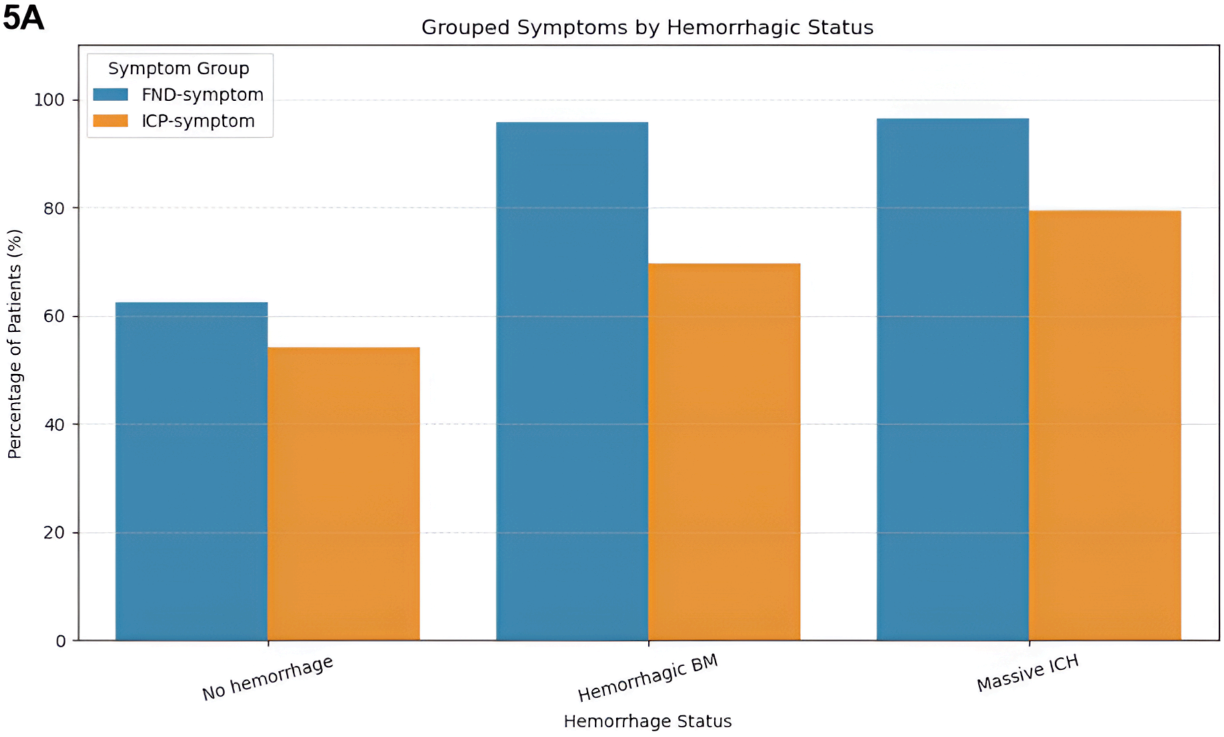

Supplementary figure 5A: Grouped Syptoms by Hemorrhagic Status.

This bar plot shows the distribution of patients with focal neurological deficits (FND) and increased intracranial pressure (ICP) symptoms across different hemorrhagic statuses: no hemorrhage, hemorrhagic brain metastases (BM), and massive ICH. The plot illustrates a higher proportion of symptoms in hemorrhagic and massive ICH cases compared to non-hemorrhagic cases.

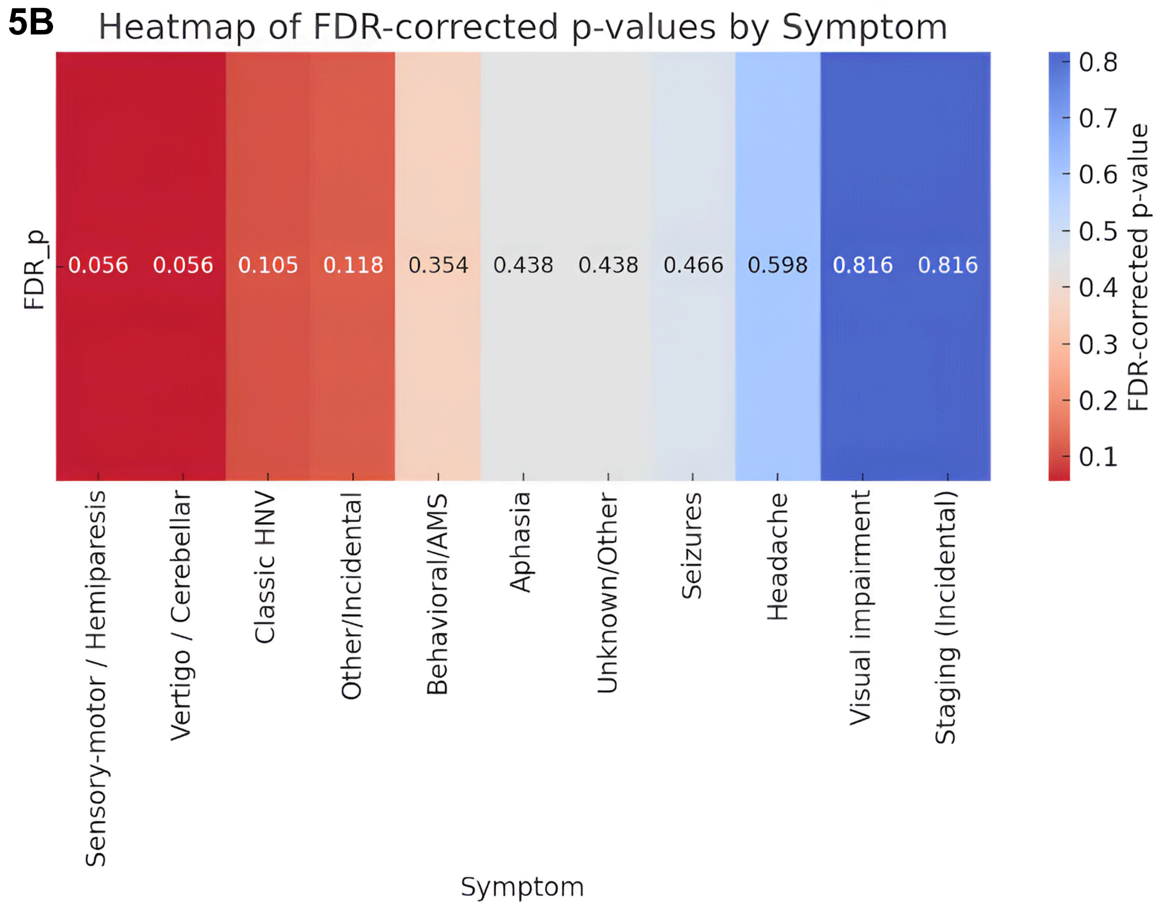

Supplementary figure 5B: Heatmap of FDR-corrected p-values for individual symptom associations across hemorrhage groups:

Colors represent adjusted significance levels from post-hoc tests comparing symptom prevalence between no hemorrhage, hemorrhagic brain metastases (BM), and massive ICH. Darker shades indicate stronger statistical significance (lower p-values). After multiple testing correction, no individual symptom varies significantly across groups, yet the statistical differences in sensory-motor deficits/ hemiparesis and vertigo/ cerebellar symptoms border significance.
